# Impedimetric DNA Biosensor Based on a Nanoporous Alumina Membrane for the Detection of the Specific Oligonucleotide Sequence of Dengue Virus

**DOI:** 10.3390/s130607774

**Published:** 2013-06-17

**Authors:** Jiajia Deng, Chee-Seng Toh

**Affiliations:** Division of Chemistry and Biological Chemistry, School of Physical and Mathematical Sciences, Nanyang Technological University, 21 Nanyang Link, Singapore 637371, Singapore; E-Mail: deng0047@e.ntu.edu.sg

**Keywords:** biosensor, impedance, membrane, nanochannel, nanopore, dengue, infectious disease

## Abstract

A novel and integrated membrane sensing platform for DNA detection is developed based on an anodic aluminum oxide (AAO) membrane. Platinum electrodes (∼50–100 nm thick) are coated directly on both sides of the alumina membrane to eliminate the solution resistance outside the nanopores. The electrochemical impedance technique is employed to monitor the impedance changes within the nanopores upon DNA binding. Pore resistance (*R*_p_) linearly increases in response towards the increasing concentration of the target DNA in the range of 1 × 10^−12^ to 1 × 10^−6^ M. Moreover, the biosensor selectively differentiates the complementary sequence from single base mismatched (MM-1) strands and non-complementary strands. This study reveals a simple, selective and sensitive method to fabricate a label-free DNA biosensor.

## 1. Introduction

Dengue fever is an infectious tropical disease caused by the dengue virus, the usual vector of which is the mosquito *Aedes aegypti* [1]. The four dengue viruses are recognized in over 100 countries and territories, and the worldwide annual infection rate is estimated to be between 50 and 100 million infections per year [2]. Hence, fast and reliable detection of dengue virus is urgently needed for management of the disease particularly during events of epidemics. To date, a variety of methods have been developed for the detection and identification of dengue virus, such as enzyme-linked immunosorbent assay (ELISA) and the polymerase chain reaction (PCR) test [3,4]. However, these methods are labor-intensive and time-consuming when large numbers of clinical samples are involved. The early and rapid diagnosis of virus is essential for epidemic prevention, and the detection of viral nucleic acid sequences based on biosensor can greatly reduce the assay time. Recently, increasing interest has focused on the application of nanoporous membranes in nucleic acid detection due to their high surface area that enhances the signals corresponding to interaction between solutes and surfaces including biomolecule reactions [5,6]. Wang *et al.* developed a DNA biosensor based on single-stranded DNA (*ss*-DNA) probe functionalized aluminum anodized oxide nanopore membranes to detect *Escherichia coli* O157:H7 DNA. The dynamic polymerase-extending (PE) method was applied in the hybridization process which improves the sensitivity of biosensor with a detection limit of 0.5 nM [7]. Smirnov's group has used a hydrothermal treated alumina membrane with shrunken nanopores for DNA sensing. Ionic conductance through shrunk nanopores was monitored during probe DNA immobilization and subsequent hybridization [8]. Li *et al.* have proposed a method for label-free DNA analysis by measuring the DNA-morpholino hybridization hindered diffusion flux of Fe(CN)_6_^3−^ probe ions through nanochannels of a porous anodic alumina membrane [9]. Our group recently reported a dengue virus particle and nucleic acid biosensor based on a sub-micrometer thick nanoporous alumina membrane coated onto a platinum disk electrode surface [10,11]. The same nanoporous alumina membrane-based biosensor was developed for the detection of *Legionella pneumophilla* DNA. This biosensor showed ultrasensitive electrochemical detection of complementary *Legionella pneumophilla* DNA ranging from 10^−13^ to 10^−6^ M with detection limit of 3.1 × 10^−13^ M [12].

Herein we employ an anodic aluminum oxide membrane as the sensing platform for the detection of the genomic sequence of dengue virus. So far, various simpler and faster techniques have been developed for DNA hybridization detection, including electrochemistry [13–15], fluorescence [16,17], surface plasmon resonance spectroscopy and quartz crystal microbalance [18–21]. Among these methods, the electrochemical technique has attracted great attention in recent years due to its low cost, simple instrumentation, and fast response time. In comparison, electrochemical impedance spectroscopy (EIS) has been a more powerful technique for DNA detection [22,23], as the resistance and capacitance values are the sensitive indicators for the changes of the surface properties which can be determined by modeling of the electrochemical data. The modeling procedure uses electrical circuits built from components such as resistors and capacitors to represent the electrochemical behavior of the modification layer and the electrode surface. Furthermore, impedance spectroscopy is a non-destructive technique and so can provide time dependent information about the ongoing processes.

Therefore, in this work, we demonstrate the use of a free-standing anodic aluminum oxide membrane for the detection of the oligonucleotide sequence of dengue virus using electrochemical impedance spectroscopy. Here, ultra-thin platinum layers are deposited as porous electrodes on both sides of the membrane and used as the working and counter electrodes, respectively, for impedance and differential pulse voltammetry (DPV) measurements in a two-electrode scheme. Presently, the approach to DNA membrane sensor is using two external Ag/AgCl or inert electrodes to measure ionic conductivity across the membrane in a two compartment cell [7,9,22]. Our approach uses ∼50–100 nm thick platinum electrodes that are coated directly on both sides of the alumina membrane as electrodes to eliminate the solution resistance outside the nanopores. In addition, the target solution can be directly applied in small quantity of only 30 mL in volume onto one side of the membrane without need for a cell assembly setup, which greatly simplifies the whole sensing procedure and reduces sample volume by a few thousand-fold. Probe DNA sequences are covalently attached within the pores of the nanoporous alumina membrane using glutaraldehyde cross linking [24]. Binding of target DNA to the probe inside nanopores causes impedance changes due to blocking of nanopores which provides the sensing signals.

## 2. Experimental Section

### 2.1. Materials and Instruments

Nanoporous alumina membranes (Anodisc™, 13 mm diameter, 20 nm and 200 nm pore sizes) were purchased from Whatman (Maidstone, Kent, UK). potassium hexacyanoferrate(II) trihydrate (>99.5%) was purchased from Merck (Singapore). 3-aminopropyltrimethoxysilane (APS), glutaraldehyde, sodium chloride, urea, potassium hexacyanoferrate(III) and phosphate buffer saline (PBS, 1×) were obtained from Sigma-Aldrich (Singapore). tris(Hydroxymethyl)-aminomethane was purchased from Bio-Rad Laboratories (Singapore). KCl was from Sinopharm Chemical Reagent Co. Ltd. (SCRC, Shanghai, China). Ultrapure water from a Sartorius Ultrapure Water System (Gottingen, Germany) was used for all preparations.

All DNA oligonucleotides were synthesized by Sigma-Aldrich. DNA probe sequence of Dengue virus (3′-GGG CAG AAA AAG GCA ACC AAC AAG TAG TCT CCA ATG GCG (CH_2_)_6_NH_2_-5′), complementary target sequence (5′-CCC GTC TTT TTC CGT TGG TTG TTC ATC AGA G-3′), single base mismatch target sequence MM-1 (5′-CCC GTC TTC TTC CGT TGG TTG TTC ATC AGA G-3′) and 21 bases mismatch target sequence MM-21 (5′-CGC CTT TTT CCG TTG GTT ATT CAT CAG AGA T-3′). All target analyte DNA solutions were prepared using 1.0 M TRIS buffer pH 7.0.

### 2.2. Sputtering of Platinum Electrodes on Opposite Side of the Alumina Membrane

An ∼50–100 nm thick platinum film was directly sputter-coated on both sides of the alumina membrane (20 nm pore size) using JEOL JFC-1600 Auto Fine Platinum Coater (Tokyo, Japan). The Pt coating was performed using a current of 20 mA for 600 s. A 1 mm width space along the edge of the AAO filter was left uncoated on both sides to avoid short circuit. The corresponding electrodes on opposite sides of the membrane were used as the working and counter electrode respectively. Aluminum tapes were placed in electrical contact with the platinum coated alumina membrane which can be connected to a potentiostat for impedance and DPV measurements.

### 2.3. Covalent Immobilization of DNA Probes into Alumina Nanopores

**Figure 1.**
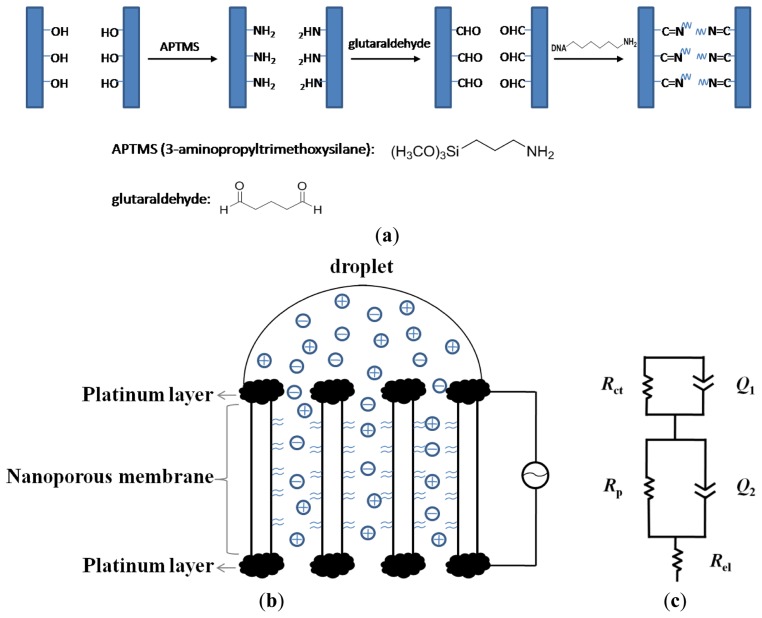
(**a**) Scheme of DNA immobilization procedure; (**b**) Schematics of nanoporous alumina membrane for impedimetric biosensing of DNA target; (**c**) The equivalent circuit model for fitting the impedimetric experimental data.

The alumina membranes were first immersed in 5% APS ethanol solution for 1 h. After washing with ethanol, the membranes were baked at 120 °C for 3 h to ensure the completion of covalent linkage of silanes to the surface. The remaining modifications were performed after deposition of platinum films on the membrane. After platinum coating, the surface of the alumina membrane was activated by overnight treatment in 7.5% aqueous solution of glutaraldehyde, followed by thorough washing with ultrapure water and drying with nitrogen gas. Approximately 30 μL of ∼100 μM solution of aminated nucleic acid was added onto the surface and kept at high humidity overnight. After that, the membrane was washed with 1 M NaCl and with copious amounts of DI water and then dried with nitrogen. A scheme of this procedure is shown in Figure 1(a).

### 2.4. Fabrication of Biosensor Array and Electrochemical Measurement

The membrane probe, with an effective area of 0.8 cm^2^ was placed horizontally, with the upper side of the membrane being used as the working electrode, and the nether side of the membrane as the counter electrode, as shown in Figure 1(b). An amount of 50 μL of 5.0 mM Fe(CN)_6_^3−/4−^ in 1 × PBS solution was only dropped onto the surface of the working electrode. Impedance measurements were performed with autolab potentiostat over a frequency range of 0.01 to 10^5^ Hz under small AC voltage (5 mV). Electrochemical behaviour of the biosensor was characterized using DPV technique in the presence of 1.0 mM Fe(CN)_6_^4−^ in 1 × PBS solution. DPV was carried out using 200 ms pulse width, 50 mV pulse height, pulse period of 500 ms and potential increment of 4 mV.

### 2.5. Procedure for DNA Targets Sensing

Hybridization was typically performed by adding DNA target solution onto the membrane surface at room temperature for 1 h. The unhybridized DNA was washed off from the membrane by copious amounts of ultrapure water, followed by electrochemical measurements at room temperature.

### 2.6. Regeneration of DNA Biosensor

The regeneration of DNA sensing device was achieved by treatment with 9 M urea, which included 1 h soaking and washing with copious amounts, followed by ultrapure water rinsing.

## 3. Results and Discussion

### 3.1. Characterization Using Differential Pulse Voltammetry (DPV)

**Figure 2.**
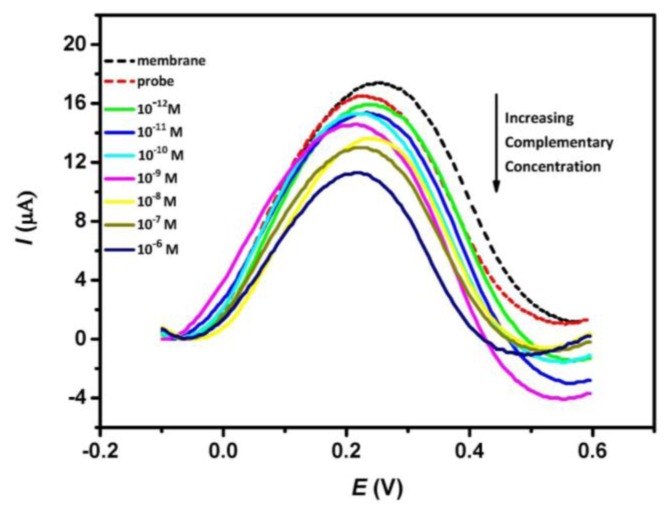
Differential pulse voltammetry current signal response of the integrated nanoporous membrane sensor toward increasing concentration of complementary DNA target from 10^−12^ to10^−6^ M.

Figure 2 shows the typical DPV peak currents of nanoporous alumina membrane, DNA probe-modified membrane and subsequent treatment of the DNA probe-modified membrane with different concentrations of complementary *ss*-DNA solution. DPV experiments were performed in the presence of 1 mM Fe(CN)_6_^4−^, 1× PBS buffer (pH 7.0). It is observed that the differential oxidative peak currents decrease after the immobilization of the probe DNA in the membrane. This is attributed to the blocking effect caused by the probe DNA which restricts the mass transfer of redox species along the nanopores. Subsequent treatment of the modified membrane with complementary target DNA leads to further decrease in the oxidative peak current and a successive drop in the peak current is observed with increasing concentration of complementary analyte. This change indicates that the hybridization events influence the diffusion of redox species through the nanopores and the decrease in current is presumably related to the amount of DNA duplex formed inside the nanopores. Insignificant peak shifts can also be observed in Figure 2. This is attributed to a consequence of nonspecific adsorption of DNA sequence onto the electrode surface which affects the electron transfer process between the solution ferrocyanide and the ultra-thin platinum layer electrode.

### 3.2. Impedimetric Sensing of DNA Targets

**Figure 3.**
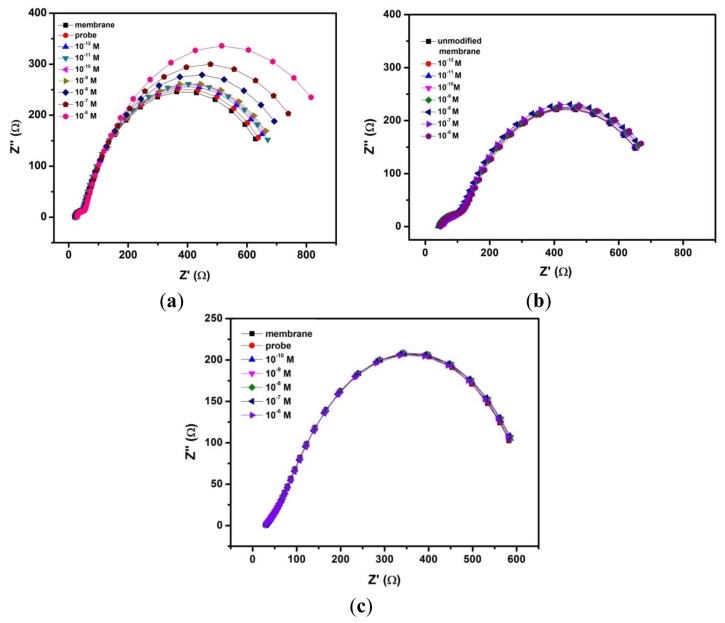
Nyquist plots of (**a**) integrated nanoporous membrane electrode grafted with DNA probe, (**b**) unmodified nanoporous membrane electrode in response toward increasing concentrations of target DNA (10^−12^∼10^−6^ M), (**c**) 200 nm pore size membrane sensor grafted with DNA probe toward increasing concentration of DNA targets (10^−10^∼10^−6^ M).

EIS can provide useful information on the changes in the physical structures of the nanopores upon chemical grafting with DNA probes. Further, it can distinguish the individual contributions of capacitive, Faradaic and diffusive components under investigation. Figure 3(a) shows the impedance spectra (presented in Nyquist plot format) of unmodified nanoporous membrane electrode, membrane grafted with probe DNA and subsequent treatment with complementary *ss*-DNA targets from 10^−12^ to 10^−6^ M sample solutions. Hybridization with increasing concentration of complementary target DNA clearly brings about an increase in the overall impedance of the system, thereby enlarging the incomplete semicircle of the Nyquist plot (Figure 3(a)). Unmodified integrated membrane sensor was also tested against different concentrations of target DNA (Figure 3(b)), no significant changes can be seen in the overall impedance. In addition, we carried out the same procedure for a probe modified 200 nm membrane which however, gives no response toward complementary DNA targets (Figure 3(c)). These confirm that the smaller 20 nm pore size together with a specific probe are needed in order to achieve changes in the sensor's signal response arising from impedance change by the blocking effect of the DNA.

To accurately analyze the EIS data, the experimental data was fitted to the equivalent circuit (Figure 1(c)) by model fitting software and summarized in Table 1. The optimal equivalent circuit consists of five elements: charge transfer resistance of the electrode reaction *R*_ct_, in parallel with a constant phase element (CPE1) which represent the double layer capacitance *C*_dl_, the resistance of pores *R*_p_, in parallel with another constant phase element (CPE2) which represent the pore capacitance *C*_p_, and the electrical resistance of the electrode *R*_el_. Placing the electrodes directly at the membrane eliminates the contribution from the resistance of solution outside the pores. This can be extremely useful for direct sensing of DNA in complex matrices such as blood which contains significant amounts of micrometer-sized and sub-micrometer sized cells, cell fragments and large protein aggregates and these can be readily excluded from the membrane nanopores during impedimetric measurements. Also, since microliter amount of the sample solution is added to the working (upper) electrode of the membrane probe, while the electrode on the nether side remains dry, the electrochemical conduction can occur only via the nanopores. Thus, the membrane sensor can sensitively monitor changes of current arising from ionic conductance within the nanopores which otherwise, is not possible in a non-membrane configuration. In this case, EIS can clearly distinguish this ionic conductance or pore resistance from the Faradaic and capacitive reactions that occur at the electrode-electrolyte interface. Because the DNA hybridization occurs within the nanopores and can affect the mass transfer of ions within the nanopores, thus we focus on the change of the pore resistance value (*R*_p_) which ought to reveal relevant information about the nature of the DNA target and its quantity.

As can be seen from Figure 3(a), the fitted curves show very good agreement with the experimental EIS spectra presented in the Nyquist plot format. The value of *R*_p_, obtained from the curve fitting, for the unmodified nanoporous alumina membrane is 667.3 ± 17.4 Ω. Upon the immobilization of probe DNA strand in the nanopores, *R*_p_ value increases to 690.5 ± 14.4 Ω due to the electrostatic repulsion between the negatively charged DNA and the redox couple Fe(CN)_6_^3−/4−^. Moreover, after subsequent treatment with increasing concentrations of complementary *ss*-DNA from 10^−12^ to 10^−6^ M, further increases are observed in the *R*_p_ value from 715.2 ± 17.9 Ω to 867.8 ± 30.6 Ω. The change in the pore resistance *R*_p_ is the most significant among all the electrical elements presented in the equivalent circuit, thus indicating that the mass transfer of ions is influenced by the DNA hybridization events within the nanopores. This phenomenon can be attributed to the “volume exclusion” mechanism as postulated by several reports [7,8,22,25], in which the formation of DNA duplex structure can decrease the effective cross-section of the nanopores, and prevent the redox species from reaching the electrode on the nether side. It is however, unclear if this ‘volume exclusion’ arises from the effect of charge repulsion between the negatively charged DNA and the redox probe Fe(CN)_6_^4−^ or from the impeded diffusive mass transfer of redox probe when the rigid DNA duplex extends outward from the nanochannel walls, forming a thicker DNA monolayer.

### 3.3. Analytical Performance

**Figure 4.**
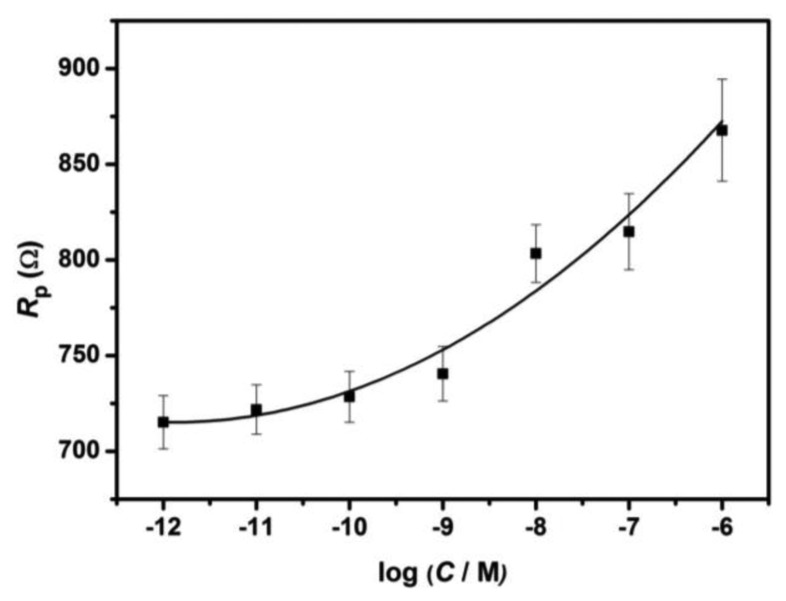
Calibration plot of pore resistance against the logarithm of complementary target DNA concentration.

The pore resistance increase is determined by the surface coverage of immobilized probe DNA and the amount of its hybridization with target DNA [25], as indicated by Table 1 and Figure 4. The pore resistance increases linearly with the increasing concentration of target complementary DNA upon hybridization in the range of 10^−12^ to 10^−6^ M (*R*^2^ = 0.96). The detection limit was found to be 2.7 × 10^−12^ M which is comparable to the reported AAO membrane-based impedimetric sensor [7].

### 3.4. Selectivity Experiment

**Figure 5.**
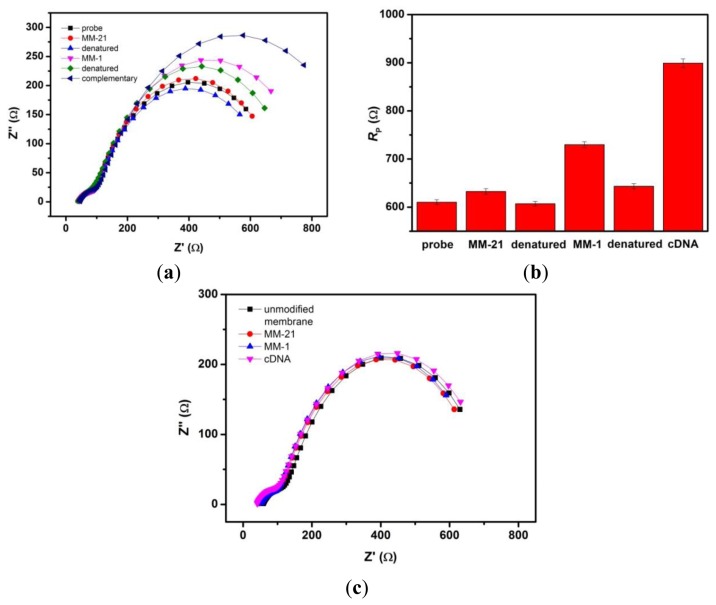
(**a**) Nyquist plots of ssDNA probe grafted nanoporous alumina membrane after exposure to noncomplementary target sequence (MM-21), single base mismatched target sequence (MM-1) and complementary target sequence. Each measurement step was followed by a denaturing step; (**b**) Bar chart illustrating the *R*_p_ values obtained from the impedance responses of the integrated nanoporous membrane sensor after consecutive steps of incubation with DNA targets followed by a regeneration step; (**c**) Nyquist plots of unmodified nanoporous alumina membrane in response to noncomplementary target sequence (MM-21), single base mismatched target sequence (MM-1) and complementary target sequence, following the same procedure and order as (a).

The selectivity of nanoporous alumina membrane-based DNA biosensor was tested by doing different reference assays. Denaturation (or regeneration) procedure was carried out between the detection steps of the non-complementary and complementary sequences by using 9.0 M urea after each step of hybridization to eliminate the carryover effect. Figure 5(a) shows Nyquist Plots of this biosensor towards successive exposure to a non-complementary 31-mer target sequence with 21 bases mismatch (MM-21), target sequence with single base mismatch (MM-1), and complementary target sequence generally found in dengue virus. Each measurement step was followed by the regeneration step. All target analyte concentrations used were 10^−6^ M. Figure 5(b) shows the *R*_p_ values obtained from the impedance data after fitting. After incubation of the biosensor with MM-21 DNA strands, insignificant change was observed. Subsequent exposure to MM-1 DNA sample, the *R*_p_ comparatively increased. After incubation in complementary target solution, the interaction of probe and complementary target yielded a significant change in *R*_p_. These results clearly demonstrate the good selectivity of this biosensor. As a control, we carried out the same experimental procedure using an unmodified nanoporous membrane sensor. Figure 5(c) shows the relatively unchanged response of unmodified nanoporous membrane towards noncomplementary target sequence (MM-21), single base mismatched target sequence (MM-1) and complementary target sequence.

It is well known that single-nucleotide mismatches in normal dsDNA will affect the local environment of adjacent bases. This effect is dependent on the identity of the base and the position of the mismatch within the DNA helix. A possible interpretation of the dramatic difference in impedance between single mismatched target DNA and complementary target DNA is that the single base mismatch positioned within the DNA sequence can cause kinks during the binding. These kinks will bend the DNA duplex toward the surface of inner wall, resulting in a decrease in the thickness of DNA monolayer along the nanochannel walls. This is unlike the complementary DNA duplex which is expected to extend fully away from the nanochannel wall, so to give a thicker DNA monolayer that can impede the movement of redox species in the nanochannel.

Finally, we also measure the reproducibility of the sensor response for three different membrane sensors. Different membrane sensors do not give the same *R*_p_ values at a particular DNA concentration, but when normalized against the blank signal *R*_p,0_ (obtained in the absence of DNA target), the normalized signal is highly reproducible, giving an error of 0.5% to 1%, at low concentration range from 10^−12^ to 10^−10^ M.

## 4. Conclusions

In this work, we demonstrate that platinum sputter-coated onto both sides of a nanoporous alumina membrane can be used as a micrometer-thick membrane probe for the high performance sensing of DNAs. Firstly, this ultrathin single membrane-based sensor is simpler than the usual metal or carbon electrodes in which the overall sensing platform is an integrated probe, instead of the usual three electrodes system. Besides, the 50–100 nm thick layer electrodes are positioned close to the nanopores so that the electrolyte resistance outside the membrane has minimal effect on the overall impedance measurements. Thus, the fitted pore resistances are highly useful for quantitative and specific detection of complementary target sequence and encounter minimal interferences from the sample matrix outside the membrane. The nanoporous alumina membrane-based impedimetric DNA biosensor shows a considerably low detection limit (2.7 × 10^−12^ M) of 31-mer complementary analyte. In addition, the biosensor selectively differentiates the complementary sequence from target sequences with 21 bases mismatch (MM-21) and single base mismatch (MM-1). Overall, this integrated membrane probe is potentially useful for the development of inexpensive, selective and sensitive DNA biosensors for Dengue.

## Figures and Tables

**Table 1.** Fitting parameters using the equivalent circuit model.

	***R*_ct_/Ω**	***Q****_1_***/Ω^−1^s^n^**	***n*_1_**	***R*_p_/Ω**	***Q*_2_/Ω^−1^s^n^**	***n*_2_**	***R*_el_/Ω**
Bare membrane	59.9 ± 7.2	0.0035 ± 0.0004	0.42 ± 0.02	667.3 ± 17.4	0.0048 ± 0.0001	0.79 ± 0.01	20.7 ± 0.3
Probe immobilization	50.6 ± 3.9	0.0027 ± 0.0002	0.48 ± 0.01	690.5 ± 14.4	0.0048 ± 0.0001	0.78 ± 0.01	17.9 ± 0.2
*c*DNA a 10^−12^M	59.7 ± 6.6	0.0032 ± 0.0003	0.43 ± 0.01	715.2 ± 17.9	0.0045 ± 0.0001	0.78 ± 0.01	19.2 ± 0.3
*c*DNA 10^−11^M	47.2 ± 4.1	0.0025 ± 0.0003	0.45 ± 0.02	721.8 ± 16.9	0.0046 ± 0.0001	0.76 ± 0.01	20.3 ± 0.3
*c*DNA 10^−10^M	53.0 ± 5.6	0.0031 ± 0.0004	0.43 ± 0.02	728.5 ± 17.4	0.0045 ± 0.0001	0.77 ± 0.01	22.0 ± 0.3
*c*DNA 10^−9^M	50.7 ± 5.8	0.0035 ± 0.0004	0.43 ± 0.02	740.5 ± 18.4	0.0048 ± 0.0001	0.77 ± 0.01	21.2 ± 0.3
*c*DNA 10^−8^M	44.9 ± 3.9	0.0024 ± 0.0003	0.47 ± 0.02	803.4 ± 19.1	0.0045 ± 0.0001	0.75 ± 0.01	20.4 ± 0.3
*c*DNA 10^−7^M	64.5 ± 9.9	0.0040 ± 0.0006	0.39 ± 0.02	814.8 ± 23.9	0.0047 ± 0.0001	0.78 ± 0.01	23.2 ± 0.4
*c*DNA 10^−6^M	120.6 ± 31.8	0.0051 ± 0.0006	0.35 ± 0.01	867.8 ± 30.6	0.0044 ± 0.0002	0.80 ± 0.01	25.7 ± 0.4

a Complementary target DNA with sequence given in Section 2.1.
